# It would feel weird to not drive my car! Exploring the role of habits in public policy acceptance of carbon taxations

**DOI:** 10.1007/s13280-024-02115-3

**Published:** 2024-12-21

**Authors:** Noah Linder, Patrik Sörqvist, Daniel Lindvall, Sverker Jagers, Stephan Barthel

**Affiliations:** 1 Global Economic Dynamics and the Biosphere, Royal Swedish Academy of Science, Lilla Frescativägen 4A, SE-104 05 Stockholm, Sweden; 2 Department of Building Engineering, Energy Systems and Sustainability Science, University of Gävle, Kungsbärcksvägen 47, SE-801 76 Gävle, Sweden; 3 Department of Health, Learning, and Technology, Luleå University of Technology, Luleå, Sweden; 4 Department of Earth Sciences, Uppsala University, Villavägen 16, 752 36 Uppsala, Sweden; 5 Department of Political Science, Gothenburg University, Huvudbyggnad Vasaparken, Universitetsplatsen 1, 405 30 Gothenburg, Sweden; 6 Stockholm Resilience Centre, Stockholm University, Stockholm, Sweden

**Keywords:** Carbon tax, Car use, Climate mitigation, Habits, Policy acceptance, Public

## Abstract

This study aims to expand the understanding of public acceptance of carbon taxes by exploring the role of habits. Habits play a pivotal role in guiding our behaviors and reasoning and can even influence our self perception and identity but remain an underexplored variable in relation to public policy acceptance. We employed a large scale (*N* > 5200) national survey to measure public acceptance of higher carbon taxation in Sweden, also capturing car driving habits, car usage, and other relevant variables. The findings show that habit strength is negatively correlated with policy acceptance, regardless of self reported driving distance, while also appearing to moderate the relationship between policy acceptance and environmental concern and political leaning, variables previously shown to be of relevance. The study suggests that the influence of habits needs to be recognized to better understand the formation of climate policy acceptance, and exploring this perspective paves the way for future research.

**Supplementary Information:**

The online version contains supplementary material available at 10.1007/s13280-024-02115-3.

## Introduction

To successfully reduce greenhouse gas emissions and to keep the global average temperature well below 2 °C above preindustrial levels, as committed to in the Paris Agreement, several experts advocate for countries to introduce carbon pricing mechanisms (Andersson 2019; Köppl and Schratzenstaller 2023). Cap and trade systems or carbon taxation are deemed to be among the most efficient policies to curb emissions (Baranzini and Carattini 2017; Best et al. 2020). The realization of such measures, however, hinges on the social acceptance of these policies, and attaining public consent for carbon taxation, i.e., public acceptance, is especially difficult (Furceri et al. 2023). Attempts to introduce or increase an existing carbon tax are infamously known to risk triggering political resistance (Douenne and Fabre 2020; Reungoat et al. 2022), which might help explain why, in most countries that have introduced pricing mechanisms, the levies have typically been set at levels well below science-based recommendations (Klenert et al. 2018; World Bank 2023).

In recent years, the research in the field of climate policy acceptance has been rapidly developing and a wide array of factors influencing attitudes to different climate policy instruments have been identified. The findings encompass factors directly related to the policy instruments themselves (i.e., “policy-specific beliefs”), such as perceived fairness or effectiveness of the instruments (Bergquist et al. 2022; Maestre-Andrés et al. 2019), but also psychological factors, such as environmental concern, ideological positioning, trust, and sociodemographic factors (Drews and van den Bergh 2016; Davidovic et al. 2020; Muhammad et al. 2021; Coleman et al. 2023).

The novel contribution of this study, however, is that it aims to unpack a variable much less explored in relationship to public policy acceptance, the role of habits, i.e., our tendencies to respond automatically to specific cues, acquired by repeating behaviors in stable contexts (Wood and Runger, 2016). This is an aspect that has basically been neglected in the literature on public policy acceptance. Habits could be highly relevant for climate policy acceptance, because to achieve a transition toward a low-carbon future, many daily routines (containing strong habitual components) linked to private transportation will need to be disrupted, from petrol- and diesel-powered vehicles, to travel with alternative and more sustainable moods of transport.

Recent research has highlighted the importance of understanding the role of habits when addressing sustainable behavior change, how to break habits, and how they influence attitudes and self-perception (Verplanken and Whitmarsh 2021; Linder et al. 2022). Studies have also demonstrated that habits and past behaviors can shape and reinforce personal values and attitudes (Adriaanse et al. 2018; Mazar and Wood 2018) and thus potentially affect acceptance of policy that is perceived as a threat to these. As a pioneering work on these associations, the study aims to explore the role of habits for the public’s propensity to accept climate policy instruments. To this end, we ask the following research questions: 1) Do self-reported car-driving habits affect the propensity to accept or resist a carbon tax increase?; 2) If so, is the effect comparable to other well-established variables, such as environmental concern and political leaning?; and 3) How do car-driving habits interact with these variables?

## Theoretical Framework

Previous research has highlighted several key variables that are related to the acceptance of carbon taxes, and in this section, we dig a bit deeper into these. A vast number of scientific studies have, for example, shown that carbon taxes are unpopular since they are perceived to have negative personal economic effects, particularly affecting vulnerable groups in society (Thalmann 2004; Hammar and Jagers 2007). There is also research, suggesting that intrinsic psychological factors influence policy acceptance, such as environmental concern (Hornsey et al. 2016; Kotchen et al. 2017; Douenne and Fabre 2020; Bergquist et al. 2022), ideological orientation (Jagers et al. 2018; Groh and Ziegler 2018; Harring et al. 2018; Davidovic et al. 2020; Sommer et al. 2020; 2021), and political or institutional trust (Hammar and Jagers 2007; Harring 2014; Fairbrother 2017; Fairbrother et al. 2019; Harring et al. 2018; Umit and Schaffer 2020). It is not entirely clear how these different psychological factors interfere with policy-specific beliefs.

A growing number of studies have concluded that climate policy support and rejection are also strongly linked to the perceptions of distributional fairness (Drews and van den Bergh, 2016; Carattini et al. 2018; Maestre-Andrés et al. 2019; Muhammad et al. 2021; Bergquist et al. 2022). Aspects related to policy design and implementation, such as environmental efficiency and earmarking revenues for specific purposes, are also of relevance for public acceptance of pro-environmental policy instruments (Groh and Ziegler 2018; Douenne and Fabre 2020). Research findings suggest moreover that people’s self-interest can influence other determinants of policy attitudes. Carlsson et al. (2011) suggested that individuals might have a self-serving bias, i.e., a tendency to reject policies that are seen to be in conflict with specific self-interests. In the case of carbon taxation, several researchers have shown that car driving is a self-interest that influences carbon tax acceptance (Groh and Ziegler 2018; Coleman et al. 2023; Fanghella et al. 2023). Car driving is usually measured by the reported distance or frequency of driving, yet less is known about whether the habits generated out of day-to-day car driving are linked to attitudes to different climate policies and carbon taxes. There are good reasons to assume that habits play an important role for climate policy acceptance in general, and for acceptance of carbon tax increases in particular, as these policies tend to affect the cost of driving, and thus conflict with car-driving habits. Habits have been shown to play a pivotal role in guiding our behaviors, reasoning, and decision-making (Wood and Runger, 2016). Research has estimated that approximately 40% of our daily behaviors are driven by habits, occurring without much conscious deliberation (Wood et al. 2002) and that they often constitute a major barrier for change (Wood and Runger, 2016). In our day-to-day lives, we develop many habits when we repeat actions in stable contexts, such as our daily commute to work or school, subsequently turning them into efficient, default modes of response (Gardner 2015; Wood and Runger, 2016). However, habits are not only a measure of repeated automatic behaviors. Recent studies have delved into the role habits that can play in shaping our identity and self-perception (Verplanken and Orbell 2003; Verplanken and Sui 2019). For example, individuals may identify themselves as “the type of person that goes by car” or “the type of person that recycles” and infer that these behaviors constitute a significant part of who they are, after examining their own habitual behavior (of, e.g., commuting to work by car or frequently visiting the recycling station close to home) (Gardner et al. 2012). It is unclear if established car-driving habits affect willingness to accept an increase in carbon taxation that could affect the individual’s daily routines. It is also unknown whether such habits interact with, or play a more significant role than, other values and social norms that are known to shape our identity. Hypothetically, restricting gasoline car driving through taxation can be experienced as an intrusion on some people’s identity going beyond mere perceived cost increases. Furthermore, seeing how habits often can act as a boundary condition between attitudes, values, intentions, and behavior (Kollmuss and Agyeman, 2002; Verplanken et al. 1998), it is conceivable that habit also moderates the relationship between, e.g., environmental concern and the acceptance of a carbon tax. Some initial research has explored the relationship between car habits and the moral motivation to limit car use (Bamberg et al. 2003; Eriksson et al. 2008; Verplanken et al. 1998; Ramos et al. 2020; Tomasdotter et al. 2023). This research highlights the potential of using context changes, such as moving into a new neighborhood, to break free of car habits. During these times of context change, we might be more open to changing behaviors, temporarily freed from environmental cues that activate old habitual responses, making it easier to follow through on moral motivations, aligning with the habit discontinuity hypothesis (Verplanken et al. 1998; Verplanken and Roy 2016).

Previous research, as discussed above, has shown that ideological orientation and environmental concern influence people’s propensity to accept carbon taxes (Hammar and Jagers 2006; Harring and Jagers 2013; Drews and van den Bergh, 2016), suggesting that climate policies should be designed with those attitudes in mind. In this context, it is relevant to explore the role of habits, as they might, on the one hand, independently explain the willingness to accept or resist carbon taxes, and, on the other, augment or alleviate the influence of these value-based variables on policy acceptance. By exploring the influence of habits in this context, a deeper understanding of climate policy acceptance can be garnered.

## Materials and methods

Ethical clearance for conducting this study has been received from the Swedish Ethical Review Authority (Dnr. 2023-01099-01).

### Survey data

Data was collected using a questionnaire comprising a large set of questions related to habits and attitudes that generated a large database of demographic, psychological, nonpsychological, and attitude-related variables (Barthel et al. 2023). A limited pretest was conducted before the survey was fielded. The survey was conducted between April 25 and September 8, 2023, and sent out as a postal questionnaire, but also enabling respondents to answer via a web questionnaire.

#### Participants

The population consisted of people living in Sweden who are between 18 and 84 years old. The sample was drawn from the population register and made as a nonproportional stratified sample in six categories. Gender and three age categories [(i) 18–34 years, (ii) 36–64 years, and (iii) 65–84 years] were observed. We also used two geographic categories consisting of two municipality group categories according to Swedish authorities (SKR): (i) major cities and their suburbs with more than 40% commuting, as well as larger cities between 200 000 and 400 000 inhabitants; and (ii) smaller towns and rural municipalities. Within each sample category, a random sample was drawn. The subsamples for the youngest age sample group were heavily overweighted based on survey experience that in most surveys this group has a lower response rate than other age groups.

The net sample (the sample minus postal returns, deceased, seriously ill, etc.) was 32 779 persons. Data collection was conducted in three waves to obtain a sufficient number of responses. In the first data collection wave, the response rate was 21%. In the second data collection wave, the response rate was 16%. In the third data collection wave, the response rate was 16%. The fact that the response rate varied between the data collection waves was arguably because the two additional data collection waves were conducted in July and August, i.e., during the Swedish summer vacation period.

A post-stratification strategy was then applied to ensure the sample was representative of the total population. After weighting the dataset based on several core demographics (municipality groups, gender, and age) to align with the Swedish population, a total of 5280 respondents were included in the dataset. Following the post-stratification processes, the dataset comprises of 48.8% women and 50.6% men (actual: 49.6% women and 50.4% men). The reported average monthly income was 45.85 thousand SEK (SD = 68.26) (actual 38,300 SEK) and the average age was 54.64 years (SD = 17.32) (actual 42 years). The average income and age were accordingly higher than the national averages. Also, the sample had a slight overweight of individuals living in rural areas (51.3%), compared to those living in an urban area (48.7%).

### Psychological scales and demographics

Here, we describe and report a subset of the scales that were included, and the results and analyses associated with these.

#### Socio-demographic variables

Participants stated their self-reported salary (in SEK/Swedish Krona), gender (female, male, other, does not want to reveal), and which municipality they lived in. The municipality variable was subsequently dichotomized into urban/rural living.

#### Car habit strength

We measured car habit strength using a shortened version of the self-report index of habit strength scale (Verplanken and Orbell 2003; Honkanen et al. 2005). Participants indicated their agreement to statements saying: “I normally take the car to work/campus or similar places”; “It would feel weird not to take the car to work/campus or similar places”; and “It is natural to me to take the car to work/campus or similar places.” Responses to each statement were made on a seven-point scale ranging from “do not agree at all” (1) to “agree completely” (7). The responses to the three statements were collapsed into a car habit index. The scale’s internal reliability was high (Cronbach’s alpha = 0.91). We also asked participants to state the distance they drive per year (in units of 10 km).

#### Resistance-to-change car habit

Resistance-to-change car use was measured by requesting participants to indicate their agreement to statements saying: “I can consider reducing my car commuting to work/campus/similar in order to reduce my carbon footprint”; “I would consider reducing my car commuting to work/campus/similar if the public transport were better and cheaper than today”; and “I would consider reducing my car commuting to work/campus/similar if I had extended permission to work or study remotely.” Responses to each statement were made on a seven-point scale ranging from “do not agree at all” (1) to “agree completely” (7). The responses to the three statements were inverted, to make the variable more intuitively consistent with its label (i.e., after the transformation, higher values represent higher resistance to change), and then collapsed into a car habit change resistance index. The scale’s internal reliability was fair (Cronbach’s alpha = 0.72). Participants who answered 1 (complete disagreement) in response to the statement “I normally take the car to work/campus or similar places” were not presented with (and consequently did not respond to) the car habit change resistance statements.

#### Self-reported driving distance

The participants were asked to estimate their total car-driving distance per year. Estimates were made with the Swedish distance unit called “mil.” One “mil” is 10 km. The average self-reported distance was notably higher in the sample (1964 mil) than in the population in general (1366 mil).

#### Environmental concern

We measured environmental concern using a shortened version of the self-report index of environmental concern (Schultz 2000). This scale includes underlying value structures about whether individuals care about themselves, other people, or all living things. Participants indicated their concern related to environmental impact on “myself,” “all people,” “my lifestyle,” “people in my community,” “animals and plants,” “future generations,” and “children.” The responses to the seven statements were collapsed into an environmental concern index. The scale had high internal reliability (Cronbach’s alpha = 0.92).

#### Political ideology

Participants indicated their political ideology orientation on a seven-point scale that ranged from strong right wing (1) to strong left wing (7) and with the middle (4) representing “neither left nor right.” The mean in our sample was 4.20, which is well balanced.

#### Higher carbon tax acceptability

Willingness to accept higher carbon taxes was assessed by requesting participants to respond to the following statement: “Carbon dioxide taxes on fossil fuels such as petrol and diesel should be higher in Sweden, in order to reduce greenhouse gas emissions.” The response to the statement was made on a seven-point scale ranging from “do not agree at all” (1) to “agree completely” (7).

### Analysis

Pearson product–moment correlation analyses were conducted to test the relationships between psychological scales. Ordinary least-square regressions were used to explore the relative strength among the predictors in their capacity to explain variance in tax policy acceptance (see Winship and Mare 1984, for a discussion of the appropriateness of using linear regression on data that can be viewed as ordinal, and see Robitzsch 2020, for a discussion of the similarity between ordinal and continuous variables). As a control, we also report the results from ordinal multiple regression analyses, since these may be viewed as more robust and valid, given that the data is measured on an ordinal Likert scale level (see Appendix).

## Results

Table 1 reports the descriptive statistics for the dependent variable (higher carbon tax acceptability) and the predictor variables (car habit strength, resistance-to-change car habit, environmental concern, and political ideology). Table 2 reports the Pearson’s correlation coefficients among these variables (see Table S1 in supplementary information for corresponding Spearman’s coefficients). The correlations indicate that the relationships between the variables were conceptually consistent with what one might expect. A higher car habit strength was associated with a higher resistance to change the habit. Furthermore, higher environmental concern was associated with a weaker car-driving habit strength, a lower resistance-to-change car habit, and with a more left-wing political orientation. Most importantly, a higher willingness to accept higher carbon taxes had a negative relationship with car habit strength and a negative relationship with resistance-to-change car habits, but a positive relationship with environmental concern and left-wing political orientation. Car-driving distance per year had a surprisingly weak relationship with policy acceptance.

**Table 1:** The table reports the means and standard deviations (SD) for the main variables. The response range was 1–7 in each variable. Car habit change resistance had a lower *N* because this variable did not include participants who indicated that they never take the car to work/campus or similar places

Variable	*N*	Mean	SD
Higher carbon tax acceptability	5225	3.63	2.03
Car habit strength	5070	3.43	2.22
Resistance-to-change car habit	2968	3.42	1.67
Environmental concern	4940	5.04	1.36
Political orientation	5242	4.20	1.63
Self-reported driving distance per year	4877	1963.75	19350.68

**Table 2:** The table reports the coefficients for the correlations (Pearson’s) among the main variables. ** Indicates *p* < 0.001, * Indicates *p* < 0.05

Variable	1	2	3	4	5
1. Higher carbon tax acceptability	–				
2. Car habit strength	− 0.37**	–			
3. Resistance-to-change car habit	− 0.36**	0.34**	–		
4. Environmental concern	0.45**	− 0.26**	− 0.48**	–	
5. Political orientation	0.41**	− 0.25**	− 0.29**	0.36**	
6. Self-reported driving distance per year	− 0.03*	0.03	0.04*	− 0.05**	− 0.03*

An ordinary least-square multiple regression analysis was conducted in order to test the predictor variables’ unique capacity to explain variance in willingness to accept higher carbon taxes (see Table S2 in supplementary information for a corresponding analysis, using ordinal multiple regression instead). Willingness to accept higher carbon taxes was selected as the dependent variable in the analysis, and car habit strength, resistance-to-change car habit, environmental concern, political ideology, and self-reported driving distance per year were selected as independent variables. Age, gender, self-reported income, and living area (rural versus urban) were also included as control variables (see the methods section for data on these variable’s distributions). The regression model explained about 33% of the variance (*R* = 0.57, *p* < 0.001). The coefficients for the individual predictor variables are reported in Table 3 (note that since we include the resistance-to-change car habit variable, the model only looks at complete data set, *N* = 2986 for this analysis).

**Table 3:** Results from a linear multiple regression analysis with willingness to accept higher carbon taxes as dependent variable. *F*(9, 2400) = 130.99, *p* < 0.001

Predictor	Standardized Beta	*t*	*p*
Car habit strength	− 0.18	− 9.75	< 0.001
Resistance-to-change car habit	− 0.09	− 4.37	< 0.001
Environmental concern	0.29	14.39	< 0.001
Political orientation	0.22	12.09	< 0.001
Self-reported driving distance per year	− 0.01	− 0.80	0.426
*Control variables*			
Age	0.02	1.13	0.261
Gender	0.04	2.36	0.018
Self-reported income	0.04	2.16	0.031
Living area (rural vs. urban)	− 0.08	− 4.56	< 0.001

Next, we explored how habits interacted with the other variables. A regression analysis with willingness to accept higher carbon taxes as dependent variable and environmental concern, car habit strength, and their interaction term as predictor variables, was conducted. The results are reported in Table 4. The significant interaction indicates that the relationship between environmental concern and willingness to accept higher carbon taxes is moderated by the strength of car habits. It should be noted that the interaction effect was relatively small but suggests that a strong car habit weakens the strength of the relationship between environmental concern and willingness to accept higher carbon taxes.

**Table 4:** Results from a multiple regression analysis with willingness to accept higher carbon taxes as dependent variable

Predictor	Standardized Beta	*t*	*p*
Environmental concern (EC)	0.46	18.30	< 0.001
Car habit strength (CHS)	− 0.12	− 2.72	0.007
EC × CHS	− 0.16	− 3.64	< 0.001

Another regression analysis with willingness to accept higher carbon taxes as dependent variable and political orientation, car habit strength, and their interaction term as predictor variables, was then conducted. The results are reported in Table 5. The significant interaction indicates that the relationship between political orientation and willingness to accept higher carbon taxes is moderated by the strength of car habits. This interaction effect was also relatively small but implies that a strong car habit weakens the relationship between political orientation and willingness to accept higher carbon taxes. Control analyses with predictor variables centered by their means (a method that controls for multicollinearity), respectively, were consistent with the reported analyses.

**Table 5:** Results from a multiple regression analysis with willingness to accept higher carbon taxes as dependent variable

Predictor	Standardized Beta	*t*	*p*
Political ideology (PI)	0.41	17.63	< 0.001
Car habit strength (CHS)	− 0.19	− 5.97	< 0.001
PI × CHS	− 0.10	− 3.09	0.002

It should be noted that these interactions were not significant in the context of an ordinal regression analysis (see Table S3 and Table S4 in supplementary information). Hence, the interactions must be treated with caution. This is the only difference, however, with consequences for the investigation’s conclusions, between the linear multiple regressions and the ordinal multiple regressions. In all other cases, the two ways of analyzing the results came to similar results.

## Discussion

This article explored if and how Swedish citizen’s self-reported car-driving habits are correlated with attitudes toward a carbon tax increase. Our findings show that the previously neglected variable (in relation to policy acceptance) “habits” is negatively linked to policy support for carbon taxes increase and that the strength of the effect is comparable to previously known key variables for policy support, such as political leaning and environmental concern. Furthermore, our results indicate that habits could influence policy support in other ways as evident from the interaction models; where habits are shown to moderate the relationship between environmental concern, political leaning, and policy acceptance, i.e., the results suggest that even if individuals have a concern for the environment or lean left politically, they still might struggle to accept a carbon tax increase if their car habits are simultaneously strong. These findings indicate that habit strength can act as a boundary condition for the validity of models to predict policy acceptance based on factors like environmental concern and political leaning. These patterns echo previous habit research, showing that strong habits often moderate the effects of factors such as intention, knowledge and attitude, and behavior (Ouellette and Wood 1998; Gardner 2015). However, it is important to note here again that these small interaction effects did not replicate in the ordinal regression analysis. Therefore, further research and replication are needed before drawing any stronger conclusions.

One obvious question that remains unanswered is whether the effect seen by habit strength is a direct or indirect effect. Potentially, the negative relationship between car habits and policy acceptance can be explained by many different interacting factors. It is, for example, conceivable that participants with strong stated car-driving habits perceive a carbon tax increase to have a greater negative (economic) impact on them. Thus, these policies would risk being perceived as going against people’s economic self-interest, which has previously been shown to influence policy attitudes (Carlsson et al. 2011). Similarly, part of the correlation could be explained by a self-serving bias, i.e., that some of our participants opposed the policy as it was perceived to clash with their own self-interests, e.g., the convenience of driving their car (Carlsson et al. 2011; Groh and Ziegler 2018; Coleman et al. 2023; Fanghella et al. 2023). This study tried to control for these factors to some extent: we found only a small and negligible relation between self-reported yearly distance driven and policy acceptance, which at least in theory should be a better measure of the monetary impact of a carbon tax than the habits measure (as distance has a direct correlation with cost). This result gives some indication that there is more to the habit variable than just perceived cost. Furthermore, habit strength was strongly related to habit change resistance (i.e., unwillingness to change even if conditions improved), suggesting that the resistance to change is not only rooted in conditions related to the transportation system that make it difficult or inconvenient for individuals to reduce their car driving. Taken together, this indicates that habit strength represents a deeper construct that goes beyond perceived negative costs and the convenience of taking the car for the individual. Although more research is needed to validate these conclusions, established psychological theories support this notion (see, e.g., Festinger 1962; Bem 1972; Gardner et al. 2012). For instance, individuals with strong stated car habits might be more likely to define themselves as “the type of person that drives a car” (Gardner et al. 2012) and could conclude that these behaviors form an aspect of their identity. This perspective is consistent with self-perception theory, as proposed by Bem (1972), which suggests that our attitudes and preferences are at times shaped by interpreting our own actions.

Of course, there are also endogeneity concerns with these results and conclusions, and the correlational nature of the study is going to leave some questions unanswered. For example, it is possible to restructure the moderation model so that the negative correlation between car habits and acceptance is moderated by environmental concern. This would perhaps also be theoretically sound as one can conceive that environmental concern would increase the chance of accepting a carbon tax—despite having strong car-driving habits. It is also possible that environmental concern makes it less likely to commute by car even if the possibility exists, i.e., reduces the chance of developing car-driving habits in the first place. However, since there are many reasons for developing car-driving habits, despite having environmental concerns or being left-leaning politically, i.e., lack of public transport, distance to work, kids in the household, cultural and social pressure, etc., we wanted to explore the moderating effect these habits had on policy acceptance. Furthermore, psychological processes such as cognitive dissonance, i.e., our tendency to justify our behavior and find logical reasons for it, even when it goes against our values, attitudes, and knowledge (Festinger 1962), highlight the fact that when we repeat behaviors, this can become a barrier in itself, i.e., individuals with strong car-driving habits could oppose an increase of the carbon tax with reasonings stemming from partly unconscious justification of their habitual behavior. Hence, even though there are various reasons for why some people develop car-driving habits, once such habits are established, they may act as a barrier for policy acceptance in several different ways.

Acknowledging the complexity of untangling habits from other psychological and environmental variables, our study does not aim to comprehensively dissect these relationships. This is a clear limitation. However, our purpose with this study was never to fully untangle these complex relations. We simply want to shine a light on the fact that habits and past behaviors seem to matter in policy acceptance and open the door for further research exploring and unpacking these relations. Previous research has reached some similar results on related topics, however, for example; Itzchakov et al. (2018) study found that while changing attitudes can influence the behavior of people without established habits, it has a limited impact on those with strong habits. Similarly, Mazar et al. (2023) demonstrated that strong habits lowered the impact mimicry and social norms had on behaviors. These studies do not look at policy acceptance per se, but it is possible that the same mechanisms are in play. Future research should aim to better control the impact of other related variables, such as perceived cost increases and self-serving biases to better understand the true effect of habits, by themselves, on policy acceptance. Finally, we note that there might be cultural differences between countries (Harring et al. 2018) and the generalizability of these results across cultures needs to be further explored.

## Conclusions

As governments all over the world are struggling to achieve their own climate targets, this study aims to assist by expanding the understanding of public acceptance of climate mitigation policies. As mentioned above, we find that car-driving habits could have a moderating effect on variables previously shown to be correlated with public policy acceptance, such as environmental concern and political orientation. The gravity of such finding would mean that even if an individual has a strong concern for the environment, she might struggle to accept a carbon tax increase if her car-driving habit is strong enough. Our results also indicate that resistance to change from car driving to more sustainable modes of transport is likely not only rooted in financial and technological conditions of the transportation system and that individual resistance to the much-needed sustainability transformations that lie ahead, may also be rooted in deep, partly unconscious, psychological constructs, shaped by past behavior and social actions. Conversely, our findings suggest that changes in car-driving habits could potentially reduce resistance to environmental policies. Albarracín et al.'s (2024) recent review of behavior change interventions is particularly relevant here, demonstrating that increasing the availability of behavioral options is one of the few effective strategies for promoting daily life changes. Specifically, then, investing in infrastructure for active mobility, rather than car use, may offer a path forward, and have shown to be a way of, for example, promoting biking habits in Copenhagen (Kaaronen and Strelkovskii 2020).

We can thus conclude that the influence of habits needs to be further recognized and studied to better understand the formation of the public’s climate policy acceptance. Our findings create a novel foundation for avenues in the scholarship of sustainability science, political science, and environmental economics, while they probably and unfortunately complicate work tasks for policymakers in governmental bodies worldwide

## Supplementary Information

Below is the link to the electronic supplementary material.

Supplementary file1 (PDF 120 kb)
