# Supplementary material for: It would feel weird to not drive my car! Exploring the role of habits in public policy acceptance of carbon taxations
Source: Ambio. 2024 Dec 21;54(4):670–9. doi: 10.1007/s13280-024-02115-3 (PMC11871190; doi:10.1007/s13280-024-02115-3)
Supplement: Supplementary file 1 — Supplementary file1 (PDF 120 kb) [file 13280_2024_2115_MOESM1_ESM.pdf]

Ambio

Supplementary Information

This supplementary information has not been peer reviewed.

Title: **It would feel weird to Not drive my car! Exploring the role of habits in public policy acceptance of carbon taxations**

Table S1. The table reports the coefficients for the correlations (Spearman's) among the main variables.

Variable	1	2	3	4	5
1. Higher carbon tax acceptability	-				
2. Car habit strength	-.37*	-			
3. Resistance-to-change car habit	-.35*	.35*	-		
4. Environmental concern	.42*	-.23*	-.43*	-	
5. Political orientation	.42*	-.28*	-.28*	.34*	
6. Self-reported driving distance per year	-.24*	.51*	.13*	-.16*	-.22*

Note: * indicates $p < .001$.

Table S2. Results from an ordinal multiple regression analysis with willingness to accept higher carbon taxes as dependent variable.

Predictor	Coefficient	*z*	*p*
Car habit strength	-.23	-10.28	< .001
Resistance-to-change car habit	-.14	-5.02	< .001
Environmental concern	.57	16.45	< .001
Political orientation	.28	11.34	< .001
Self-reported driving distance per year	< -.01	-0.67	.506

Table S3. Results from an ordinal multiple regression analysis with willingness to accept higher carbon taxes as dependent variable.

Predictor	Coefficient	*z*	*p*
Environmental concern (EC)	.65	14.97	< .001
Car habit strength (CHS)	-.31	-6.04	< .001
EC×CHS	.01	0.86	.392

Table S4. Results from an ordinal multiple regression analysis with willingness to accept higher carbon taxes as dependent variable.

Predictor	Coefficient	*z*	*p*
Political ideology (PI)	.49	15.83	< .001
Car habit strength (CHS)	-.23	-7.12	< .001
PI×CHS	-.01	-1.26	.207
